# Sexual Interference Behaviors in Male Adult and Subadult Tibetan Macaques (*Macaca thibetana*)

**DOI:** 10.3390/ani11030663

**Published:** 2021-03-02

**Authors:** Kui-Hai Pang, Amanda K. Rowe, Lori K. Sheeran, Dong-Po Xia, Lixing Sun, Jin-Hua Li

**Affiliations:** 1 School of Resource and Environmental Engineering, Anhui University, Hefei 230601, China; pangkuihai@126.com; 2 Interdepartmental Doctoral Program in Anthropological Sciences, Stony Brook University, Stony Brook, NY 11794, USA; amanda.rowe@stonybrook.edu; 3 Department of Anthropology and Museum Studies, Central Washington University, Ellensburg, WA 98926, USA; SheeranL@cwu.edu; 4 School of Life Sciences, Anhui University, Hefei 230601, China; dpxia@163.com; 5 Department of Biological Sciences, Central Washington University, Ellensburg, WA 98926, USA; lixing@cwu.edu; 6 School of Life Sciences, Hefei Normal University, Hefei 230601, China

**Keywords:** Tibetan macaques (*Macaca thibetana*), copulation harassment, copulatory duration, sexual competition, the post-ejaculatory phase

## Abstract

**Simple Summary:**

Sexual interference behaviors (interruption/harassment) by male nonhuman primates can lead copulating individuals to separate and is hypothetically a form of male–male competition for access to sexually receptive females. Tibetan macaques (*Macaca thibetana*) provide an example of male sexual interference that can be used to discuss the sexual competition hypothesis. We found male sexual interference in this species showed significant seasonal variation. Age did not affect the proportion or type of interference behaviors that a male performed, but his social status did. Dominant males more often interrupted copulations. Subordinate males more often directed harassment behaviors toward dominant males, which reduced copulation duration, especially the post-ejaculatory phase of copulation. Our results suggest that sexual interference (interruption or harassment) may be a tactic to reduce the mating success of other males by either preventing ejaculation or reducing the duration of the post-ejaculatory phase, which is critical for sperm transport and, thus, reproductive success.

**Abstract:**

Male nonhuman primate sexual interference, which includes copulation interruption and copulation harassment, has been related to reproductive success, but its significance has been challenging to test. Copulation interruption results in the termination of a copulation before ejaculation, whereas copulation harassment does not. We conducted this study using the all-occurrence behavior sampling method on sexual interference behaviors of seven adult and four subadult male Tibetan macaques (*Macaca thibetana*) in mating and non-mating seasons at Mt. Huangshan, China, from August 2016 to May 2017. Our results showed that males’ individual proportion of copulation interruption and harassment was higher during the mating season than during the non-mating season. In addition, dominant males more often performed interruption, whereas subordinate males more often performed harassment. We found no difference in the individual proportion of copulation interruption or harassment between adult and subadult males. Adult and subadult males both directed copulation interruption and harassment more often toward the mating male than toward the mating female. Lastly, the post-ejaculation phase of copulation was shorter when copulation harassment occurred than when it did not. Our results suggest that sexual interference may be an important mating tactic that adult and subadult males use in male–male sexual competition.

## 1. Introduction

A male’s reproductive success may change throughout the male’s lifetime, and the use of an effective mating strategy may assist the male in male–male mating competition [1]. A male may increase their reproduces success either by maximizing males’ own mating opportunity or disrupting competitors’ copulation [2,3]. Interference in the copulatory behaviors of mating pairs is a strategy that may limit the competitor’s mating success in nonhuman primates and other species [3,4,5,6,7,8].

Sexual interference is any form of disruptive behavior in which group member(s) influence or attempt to influence a dyad’s ongoing copulatory behaviors [3,4,5,6,7,8]. In nonhuman primates, sexual interference has been studied in several species, including stump-tail macaques (*Macaca arctoides*), chimpanzees (*Pan troglodytes*), golden snub-nosed monkeys (*Rhinopithecus roxellana*), and squirrel monkeys (*Saimiri collinsi*) [9,10,11,12]. Examples of interference behaviors include group members approaching mating males and females and vocalizing, reaching toward, and slapping at the mating male and/or female, moving around the pair, and sometimes making physical contact with them [9,13,14,15]. Sexual interference occurs in two forms: copulation interruption and copulation harassment (hereafter, “interruption” and “harassment”) [7,16,17,18].

Interruption ends the copulation and has been reported in rhesus macaques (*Macaca mulatta*), moor macaques (*Macaca maurus*), and stumptail macaques [7,19,20,21]. Previous work has shown that higher-ranked males engage in interruption more often than lower-ranked males, and interruption is hypothesized to prevent lower-ranked males from copulating [7,14,22]. This behavior, according to the sexual competition hypothesis, should increase the reproductive success of higher-ranking males by directly diminishing that of other males [7,14,16,18,23]. 

Compared to interruption, harassment is a more common form of sexual interference, apparently used to disrupt copulations. By definition, harassment does not end copulation or result in the separation of the mating pair before ejaculation [13,16,19]. Instead, harassment is hypothesized to be a strategy to decrease copulation duration, especially the post-ejaculatory phase, which is critical for sperm transport and, thus, reproductive success [7,16,24,25]. Harassment more often involves subordinate, lower-ranking males and is directed toward higher-ranking males who have a greater number of mating opportunities [7,16]. For example, subordinate male stumptail macaques have been observed to closely approach mating dominant males while slapping at or threatening them, and these behaviors affect the duration of the copulation [16,21]. This phenomenon has also been observed in Hanuman langurs (*Semnopithecus entellus*), long-tailed macaques (*M. fascicularis*), and white-faced saki monkeys (*Pithecia pithecia*) [26,27,28]. Harassment by subordinate males has been suggested to be a straightforward expression of male–male competition over access to receptive females to increase future mating opportunities (the sexual competition hypothesis) [7,16].

Sexual interference is frequently observed in male Tibetan macaques (*M. thibetana*) at Mt. Emei and Mt. Huangshan, China (especially Huangshan) [29,30,31]. Tibetan macaques live in hierarchical, multi-male/multi-female groups [32,33]. They are seasonal breeders, with their mating season extending from July to January and their non-mating season from February to June [34,35]. Ejaculatory copulations only occur during the mating season, but non-ejaculatory copulations have been observed during the non-mating season [36,37,38]. 

However, previous studies produced qualitative analyses of these behaviors based on ad libitum observations. For example, Li (1999) and Xiong (1991) and others observed through qualitative measures that sexual interference behaviors in Tibetan macaques occurred more often during the mating season, and they noted that harassment was a more common form of interference than was interruption [33,34,35,36,37,38,39]. Although interference behavior is quite common, there seems a lack of detailed reports on sexual interference in Tibetan macaques. 

In this study, we set out to analyze and explore variation in the occurrence of male sexual interference behaviors (interruption and harassment), the roles of males’ ages and social status on these behaviors, and the potential impact of sexual interference behaviors on copulation duration in Tibetan macaques across both mating and non-mating seasons.

We hypothesized that male Tibetan macaques would vary in sexual interference behaviors. Specifically, in accordance with the literature, we predicted that (1) when intrasexual competition is intense and reproductive copulation occurs, sexual interference behaviors will occur more often in the mating than the non-mating season, (2) dominant males will more often perform copulation interruption, and subordinate males will more often perform sexual harassment regardless of age in the mating season, and (3) sexual harassment will decrease copulation duration, especially the post-ejaculatory phase in the mating season.

## 2. Materials and Methods

### 2.1. Study Sites and Subjects

We conducted this research at the Valley of the Wild Monkeys in Mt. Huangshan National Reserve, Anhui Province, China (118°10′ E, 30°29′ N) [31,40]. This site consists of deciduous broadleaf and broadleaf evergreen mixed forests at altitudes from 600–1200 m [31,41]. Several wild groups of Tibetan macaques live in this area [31,42]. We focused our data collection on the Yulingkeng A1 (YA1) group (group size: 27–40 individuals), which has been provisioned and observed since 1986 [31,43]. 

During the study period, the number of individuals in the YA1 group fluctuated from 43 to 46 animals (three infants were born). We categorized individuals into adult, subadult, and immature categories. Following Li (1999), we classified males as adult if they were greater than or equal to 8 years old and as subadult if they were greater than or equal to 6 and less than 8 years old. Immature males were greater than or equal to 1 year and less than 6 years old. Infants (less than 1 year old) are dependent on their mother and are distinguished by their golden-colored natal coat. Our analysis focuses on 7 adult and 4 subadult males’ sexual interference behaviors (Table 1).

### 2.2. Behavior Definitions

The mating pattern for male Tibetan macaques at Huangshan is a single mount ejaculation, and the mating male usually exhibits a pause and a body/leg tremor at ejaculation. Following ejaculation, the male will remain in a mounted posture for up to approximately 30 s. This period is known as the post-ejaculatory phase and is hypothesized to be linked to fertilization success [7,31]. As such, we divided ejaculatory copulations into pre- and post-ejaculation phases. 

We judged ejaculation to have occurred either when (1) the male ceased intravaginal thrusting, showed muscular body spasms and rhythmical pants, and had a frowning, round mouth expression, or when (2) seminal fluid was visible on the genitalia or perineum of the male and/or female after the pair dismounted [31,32,33].

We defined male sexual interference as any behavior (e.g., slaps, stares, vocalizations) directed by an adult or subadult male toward a mating male or mating female, where the behavior appears to be disruptive of the copulation. This behavior was further divided into copulation interruption and copulation harassment in Tibetan macaques. We scored these behaviors using an ethogram (Table 2). 

### 2.3. Behavioral Data Collection

We collected behavioral data from August 2016 to January 2017 (mating season: mean ± SE = 24 ± 2.73 day/month, 1200 h) and February to May 2017 (non-mating season: mean ± SE = 17 ± 5.9 day/month, 640 h). A single observer (KHP) collected all behavioral data while 5–10 m away from the monkeys. Behavioral observation began at approximately 08:00 and finished at 17:30 each day.

Focal animals were all adult and subadult males of the group, totaling 11 individuals (Table 1). Using the all-occurrence behavior sampling method [44], we recorded copulatory behaviors and sexual interference behaviors when all individuals of a group were in view of the observer. The study site was marked with a systematic grid of reference points and divided into four zones [32,45,46,47]. This allowed us to record the position and behaviors of each animal accurately. KHP collected all occurrences of copulatory behaviors within the troop using a video camera (Sony HDX) and/or a voice-recording device (SONY ICD-AX412F).

For all copulatory events, we recorded the identities (name, age, and sex) of the mates and performers, the number of performers and direction of harassment and interruption behaviors, and the durations of the pre- and post-ejaculatory phases of the copulation. We summed pre- and post-ejaculation durations to calculate a total duration for each copulation in the mating season.

We defined the number of performers as the total number of individuals participating in sexual interference behaviors. For example, when two monkeys, A (male) and B (female), mated and individuals C, D, and E joined the ongoing copulation as performers, we scored three performers (monkeys C, D, and E).

**Figure 1:**
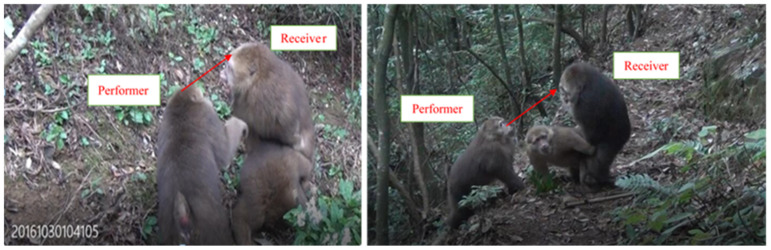
Sexual interference behaviors in Tibetan macaques (*Macaca thibetana,* performer refers to individuals performing interference; receiver refers to individuals interfered by others).

We determined the direction of interference based on the orientation of the performer’s face (e.g., performer’s face is oriented toward one or both members of the copulating pair). For example, if harassing individual C turned his face toward mating individual A, we considered C to be directing harassment towards A (Figure 1).

Based on occurrences of aggressive and submissive interactions, we used corrected normalized David’s score to assign social ranks within the group (Table 1) [48,49]. We recorded aggressive interactions as occurring when an individual stared at, chased, grabbed, or bit another individual [50,51]. We defined submissive interactions as an individual displaying a fear grin, cower, mock leave, avoid, flee, or scream in response to an aggressor [52,53]. Because mating males may be less likely to respond to aggression directed at them during copulation, we did not use agonistic interactions that occurred during copulation to calculate David’s scores.

### 2.4. Data Analysis

The same adult and subadult males (11 individuals) were present in the group during both mating and non-mating seasons. We first calculated a total proportion of sexual interference in each seasonal study period as the number of sexual interference events divided by the number of copulatory events. For example, if we recorded 15 copulatory events and 10 sexual interference events during the mating season, the total sexual interference proportion during the mating season was 10/15. We used the same method to calculate the total proportion of interruption and harassment behaviors in each season.

Additionally, we calculated the proportion of sexual interference events each individual was involved in (hereafter individual sexual interference proportion) as the number of sexual interference events performed by each individual divided by total copulations in which interference took place in each season. Similarly, we calculated the individual sexual interruption proportion and individual sexual harassment proportion.

We used descriptive statistics to quantify the following variables: total proportion of copulations with sexual interference in mating season and non-mating seasons; total proportion of occurrences of harassment and interruption in mating and non-mating seasons; and the total proportion of copulations with ejaculation. We used a one-sample Kolmogorov–Smirnov test to examine whether the individual proportions of interference, interruption, and harassment data conformed to a normal distribution (*p* > 0.05). The data were not normally distributed for each male for season, age, rank, and interference target (*p* < 0.05), so we used non-parametric statistics for these results. We used a Wilcoxon signed-rank test to compare the total proportion of interruption or harassment events during the mating and non-mating seasons. We used Spearman’s rank correlation to test the correlation between the individual proportion of sexual interference (and the individual proportion of interruption/individual proportion of harassment) and David’s scores in mating season. We used a Mann–Whitney *U* test to test for differences in the individual proportion of interruption or harassment events by adult and subadult male group members in mating season. Based on the direction of interruption or harassment, we used a Wilcoxon signed-rank test to compare the medians of sexual interference based on males’ age class (adult or subadult) and based on whether they targeted the mating male or the mating female in their interference behaviors in mating season.

We used a one-sample Kolmogorov–Smirnov test to examine whether copulation duration conformed to a normal distribution (*p* > 0.05). Since the data were normally distributed in mating season, we used a paired-sample *t*-test to compare average total copulation durations by season and pre- and post-ejaculatory total copulation durations with and without interruption or harassment in the mating season. All statistical tests were two-tailed with α set to 0.05 [54].

## 3. Results

### 3.1. Copulation Events

We observed a total of 678 copulations during the two study periods. Of the total copulations, 24 (3.54%) occurred during the non-mating season, and 654 (96.46%) occurred during the mating season. Of the total copulations, the proportion of copulations with observed ejaculation was 85.78% (561/654) compared to 14.22% (93/654) without observed ejaculation in the mating season.

### 3.2. Interference Events

We observed sexual interference in 51.92% (352/678) of copulations during mating and non-mating seasons. Harassment constituted 85.80% (302/352) of the sexual interference events, and interruption constituted 14.2% of interference events (50/352). We observed sexual interference during 53% (349/654) of copulations in the mating season and 12.5% (3/24) of copulations in the non-mating season.

### 3.3. Season and Interruption/Harassment

**Figure 2:**
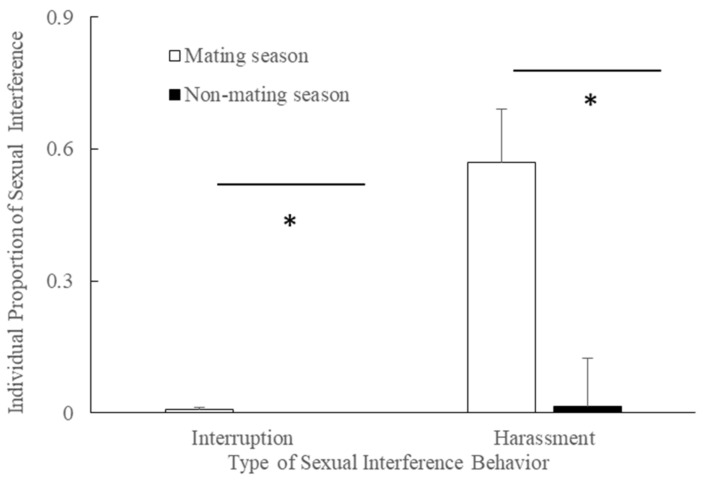
The individual proportion of interruption or harassment by adult and subadult males during the mating season and non-mating seasons (*: *p* < 0.050).

The individual proportion of interruptions by males was higher during the mating season (mean ± SE = 0.007 ± 0.240, median = 0.003) compared to the non-mating season (mean ± SE = 0.000 ± 0.000, median = 0.000, n = 11, *Z*= −2.818, *p* = 0.005 < 0.050, Figure 2).

The individual proportion of harassment by males was higher during the mating season (mean ± SE = 0.569 ± 0.124 median = 0.040) compared to the non-mating season (mean ± SE = 0.015 ± 0.011, median = 0.000, n = 11, Z= −2.193, *p* = 0.028 < 0.050, Figure 2).

### 3.4. Dominance Rank and Interruption/Harassment in the Mating Season

**Figure 3:**
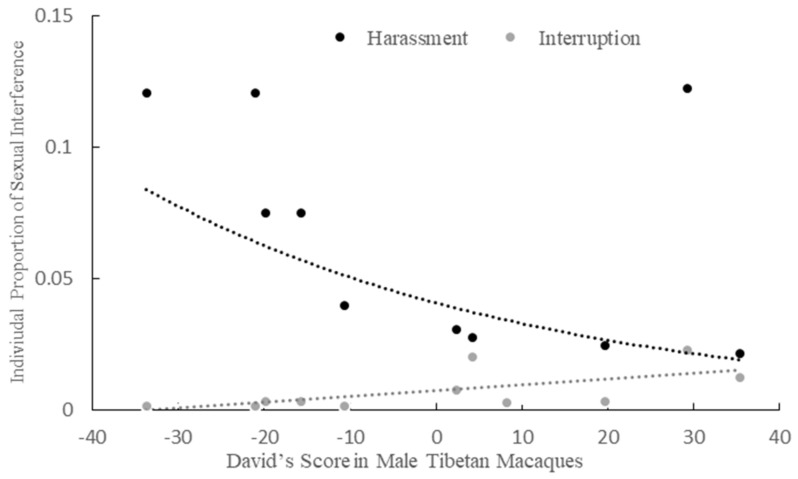
The relationship between adult and subadult male rank and the individual proportion of interruption or harassment.

We found a positive correlation between males’ dominance ranks and individual proportion of sexual interruptions (n = 11, r = 0.636, *p* = 0.035, Figure 3) in the mating season. We found a negative correlation between dominance rank and the individual proportion of harassment in the mating season. (n = 11, r = −0.336, *p* = 0.043, Figure 3).

### 3.5. Age and Interruption/Harassment in the Mating Season

**Figure 4:**
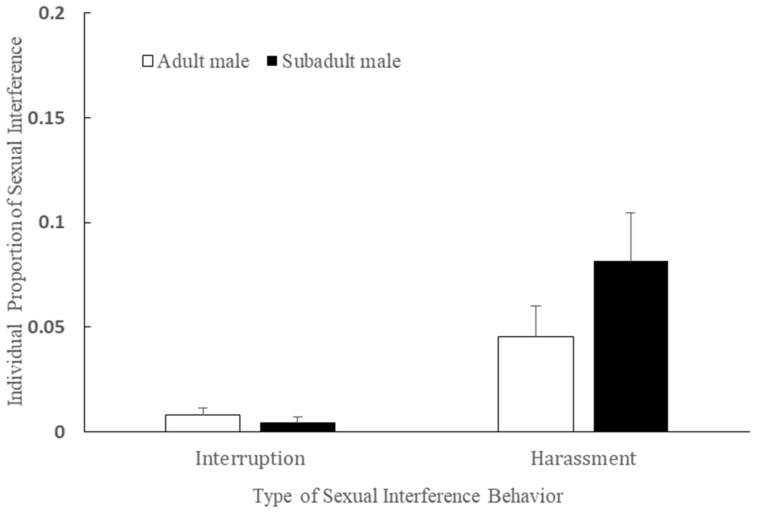
The individual proportion of interruption or harassment behaviors by adult and subadult males.

We found no significant differences in the individual proportion of interruption between adult males (n_1_ = 7, Mean ± SE = 0.008 ± 0.003, median = 0.003,) and subadult males in the mating season (n_2_ = 4, Mean ± SE = 0.004 ± 0.003, median = 0.002, *Z* = 0.578, *p* = 0.564, Figure 4).

We found no significant differences in the individual proportion of harassment between adult males (n_1_ = 7, Mean ± SE = 0.045 ± 0.149, median = 0.030) and subadult males in the mating season (n_2_ = 4, Mean ± SE = 0.082 ± 0.023, median = 0.094, *Z* = −0.856, *p* = 0.412, Figure 4).

### 3.6. Interruption/Harassment Direction in the Mating Season

**Figure 5:**
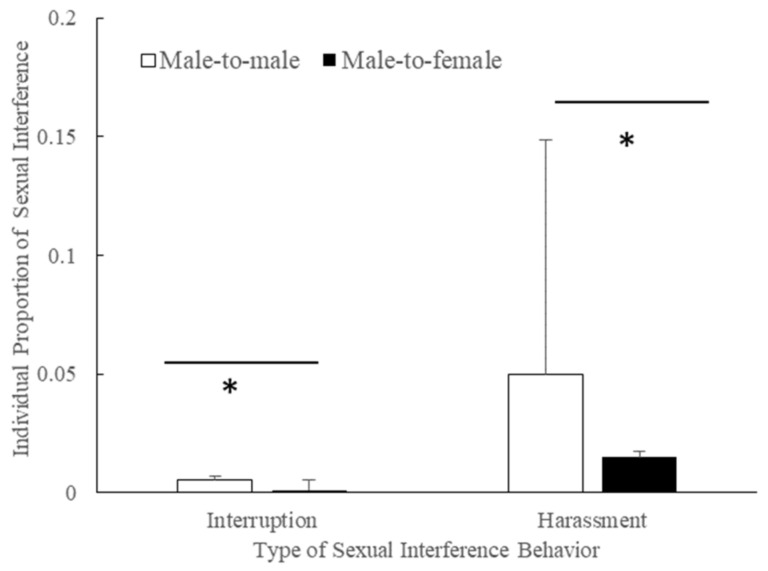
Interruption or harassment behaviors performed by adult and subadult males (Male-to-male indicates that the male harassed a mating male. Male-to-female indicates that the male harassed a mating female; *: *p* < 0.050).

Adult and subadult males directed interruptions more often toward mating males (mean ± SE = 0.006 ± 0.002, n = 11, median = 0.002) than toward mating females in the mating season (mean ± SE = 0.001 ± 0.005, median = 0.001, n = 11, *Z* = −2.536, *p* = 0.011, Figure 5).

Adult and subadult males directed sexual harassment toward mating males (mean ± SE = 0.050 ± 0.099, n = 11, median = 0.004) more often than toward mating females in the mating season (mean ± SE = 0.015 ± 0.003, median = 0.012, n = 11, *Z* = −2.934, *p* = 0.003, Figure 5).

### 3.7. Effect of Interruption/Harassment Behaviors on Copulation Duration in the Mating Season

**Figure 6:**
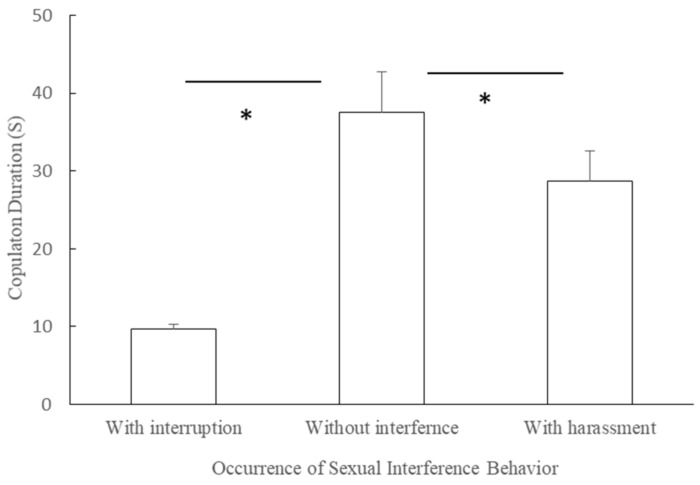
Differences in copulation durations with and without interference (*: *p* < 0.05).

The duration of copulations with interruption (mean ± SE = 9.74 ± 0.58 s, n = 11) was shorter than the duration of copulations without interference in the mating season (mean ± SE = 37.590 ± 5.230 s, n = 11, t = 5.360, df = 10, *p* = 0.0001, Figure 6).

The duration of copulations with harassment (mean ± SE = 28.75 ± 3.780 s, n = 11) was shorter than the duration of copulations without harassment in the mating season (mean ± SE = 37.590 ± 5.230 s, n = 11, t = −2.360, *df* = 10, *p* = 0.040, Figure 6).

### 3.8. Durations of Pre- and Post-Ejaculatory Phases of Copulation in Mating Season

**Figure 7:**
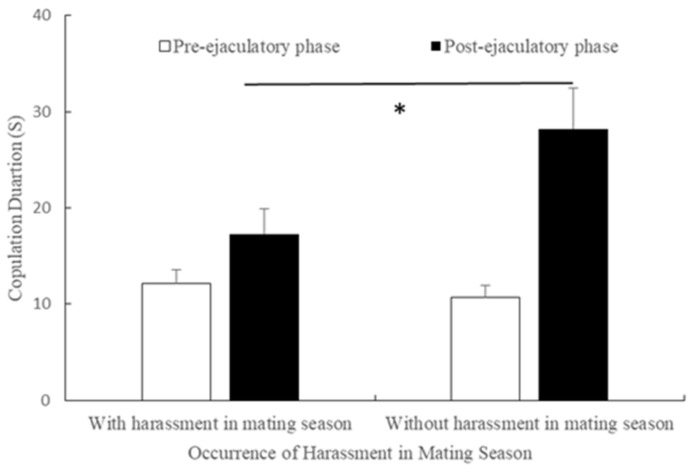
Differences in the duration of pre-and post-ejaculatory phases of mating with and without harassment in the mating season (*: *p* < 0.05).

In the mating season, the average duration of copulations’ pre-ejaculatory phase did not differ with (mean ± SE = 12.16 ± 1.460 s, n = 11) or without harassment (mean ± SE =10.75 ± 1.170 s, n = 11, t = 2.019, *df* = 10, *p* = 0.070, Figure 7). However, the average duration of the post-ejaculatory phase of copulation was shorter with harassment (mean ± SE = 17.29 ± 2.620 s, n = 11) than without it (mean ± SE = 28.23 ± 4.210 s, n = 11, t = −2.640, *df* = 10, *p* = 0.03, see Figure 7).

## 4. Discussion

Sexual interference has been observed in many nonhuman primate species [7,55,56,57], but studies focusing on the quantification of sexual interference behaviors are rare. In this study, we compared the variation in sexual interference behaviors (divided into interruption and harassment behaviors) in adult and subadult male Tibetan macaques. Our results showed that sexual interference was common in male Tibetan macaques and supported our hypothesis that variation occurs in sexual interference behaviors across adult and subadult male Tibetan macaques. We found that, as predicted, sexual interference occurred more often in the mating season than in the non-mating season. As predicted, we also found that irrespective of age, dominant males more often performed interruption while subordinate males more often performed harassment. We found that male performers directed interruption or harassment towards other mating males more often than towards mating females. Lastly, as predicted, we found that when interference (interruption and harassment) occurred, the average copulation duration was shorter compared to the average copulation duration without interference. Further, harassment decreased the duration of the post-ejaculatory phase of copulation. Although descriptive and exploratory in nature, these combined results, showing frequent interference behavior between males, suggest a potential role of interference behavior in sexual competition in our study species.

### 4.1. Seasonal Variation in Male Sexual Competition

Male sexual interference varies seasonally in nonhuman primates [28,55]. During the mating season in our dataset, there was an increase in interference (interruption/harassment). Together, these data suggest that males engaged in more sexual competition (both interruption and harassment) during the mating season when females are more likely to be fertile. Our result is consistent with the seasonal variation observed in wild tufted capuchin monkeys (*Cebus apella nigritus*) [28,58]. For that species, almost all copulation interferences occurred during the mating season, and direct copulation interruption and harassment between adult males are rare in the non-mating season [58].

Although Tibetan macaques may copulate throughout the year, births occur mainly during the non-mating season [33,34]. Thus, most conceptions occur in the mating season, and copulations outside this period occur at a time when females are likely infertile. Previous studies have shown that most females are already pregnant or lactating during the non-mating season so that most copulations occurring in the non-mating season are unlikely to be conceptive [33]. This may be why males engage in less sexual interference when copulations occur in the non-mating season because fertilization of females is unlikely. In addition, although sexual activity also occurs during the non-mating season, adult males’ testosterone concentrations decrease significantly during the non-mating season, which may also reduce male sexual interference during the non-mating season [59,60,61].

### 4.2. Effect of Male Rank on Sexual Interference in Mating Competition

Sexual interference by adult males ends in one of two ways, depending upon the relative dominance ranks of the individual performing interference and the mating individuals [7]. In the mating season, subordinate males perform harassment more than interruption, which suggests that the lower-ranking male may be constrained from directly interrupting competitors’ copulation throughout the mating period. Our study is consistent with previous findings that show that the outcome and direction of sexual behavior are correlated with the dominance rank of the individual performing interference [14,16,62,63].

It has been suggested that one of the benefits of copulation interruption by dominant males is decreased mating success of subordinate males through prevention of or reduction in competitors’ mating success before ejaculation [64]. However, interruption probably did not significantly influence the probability of future copulation by lower-ranking males in Tibetan macaques. Most of the successful copulations by subordinate males during the mating season occurred away from the group, as either sneak copulations or copulations timed to coincide with those of the higher-ranking males [29,31].

In contrast, although harassment by subordinate males rarely prevented ejaculation or interrupted copulation, it nonetheless could reduce reproductive success by reducing the post-ejaculatory period [16,65]. Some subordinate males more frequently harassed mating males than mating females in this population of Tibetan macaques. A similar pattern of harassment direction was found in langurs and male Japanese macaques (*M. fuscata*) [7]. Bruce (1992) argued that dominant males ended copulations much more quickly when confronted with harassment by subordinate males [7,16]. Harassment could indirectly benefit the mating success of subordinate males by drawing the attention of the alpha male to interrupt the copulation of a mating male.

### 4.3. Effect of Interference Direction in Mating Competition

Harassment direction by males may be a form of sexual competition in the mating season, during which the mating male was more often the target of aggression from harassers than was the mating female [66,67,68]. Our study results are consistent with other research showing that the mating male is harassed more than the mating female [7]. Intra-sexual aggression during copulation harassment has also been reported in bonobos (*Pan paniscus*) and *Cebus apella* [66,69]. This pattern has been interpreted as an expression of sexual competition in the mating season [69].

However, aggression during mating interference followed a pattern similar to overall male–male aggression [32]. An alternative explanation is that the motivation for male harassment direction may be similar to retaliation [16] because it also provides subordinate males a relatively safe opportunity to retaliate for previous male–male aggression [16,70,71,72]. In addition, mating males remain quietly intromitted following ejaculation in the post-ejaculatory phase and, therefore, could be more vulnerable to attack from others and less likely to behave aggressively [16].

In macaques and chimpanzees, mating aggression by the male engaged in interference is sometimes directed toward the mating female so that risks may be incurred by the mating male or the mating female. Punishment of the mating male and mating female may also occur sometime after the observed copulation [7,70,71,72].

### 4.4. Post-Ejaculatory Phased and Sperm Transport

Niemeyer and Chamove (1983) presented quantitative data demonstrating that interruption before ejaculation successfully prevented ejaculation. They also stated, but provided no quantitative data, that harassment shortened the post-ejaculatory phase of mating males. The data we collected provide empirical support for their observation. If sperm transport is facilitated in part by the length of the post-ejaculatory phase and, thus, ultimately affects reproductive success [7], then harassment may, in fact, reduce the reproductive success of mating males.

In contrast to their rare participation in interruption, lower-ranking males in our study population were active in harassment during the post-ejaculatory phase [33], which may ensure that competitor males’ sperm is quickly displaced by other males after copulation. In addition, the duration of the post-ejaculatory phase may be critical to fertilization as it influences sperm transport. Thus, harassment that leads to a reduction in the duration of this phase could permit a female to solicit copulations from other males [7,25].

Transcervical sperm transport in rats required 6–10 min after ejaculation or completion, and even a single intromission 2 min after ejaculation could dislodge the vaginal plug and disrupt sperm transport [73,74]. Bruce and Estep (1984) speculated that male pigtail macaques (*M. nemestrina*) may interfere with one another’s sperm in much the same way [75]. A shorter post-ejaculatory phase may influence sperm transport and, therefore, may decrease reproductive success in Tibetan macaques.

## 5. Conclusions

In conclusion, our results suggest that sexual interference in a hierarchical, multi-male/multi-female primate social group may be a male intra-sexual competition strategy. We could not provide direct evidence that sexual interference reduced the likelihood of conception of the mating males and increased mating opportunity and success of the harasser, but our results show that copulation harassment shortens the duration of the post-ejaculation phase of copulations, which likely leads to lowered rates of conception. Our study is foundational in showing the potential importance of sexual interference for intra-sexual competition in Tibetan macaques. Future studies can build from our findings to provide data predictive of conception success in both the harasser and harassed individuals.

## Figures and Tables

**Table 1:** Age class and rank of the males in the Yulingkeng A1 (YA1) group during the study period. (DS means David’s Score).

Age Class	Rank (DS Score)	Male	Immigrate Date/Birth Date
subadult male	1 (35.3)	HXM	2010-02-23 (Birth Date)
adult male	2 (29.18)	TG	2003-??-?? (Immigrate Date)
adult male	3 (19.71)	ZB	2013-08-?? (Immigrate Date)
adult male	4 (8.21)	GS	1984-??-?? (Immigrate Date)
adult male	5 (4.18)	YRB	2008-01-22 (Birth Date)
adult male	6 (2.3)	BT	2011-02-?? (Immigrate Date)
adult male	7 (−10.7)	HM	2014-11-?? (Immigrate Date)
adult male	8 (−15.7)	DS	2013-08-?? (Immigrate Date)
subadult male	9 (−19.8)	TRG	2010-04-24 (Birth Date)
subadult male	10 (−21)	YCLO	2010-06-21 (Birth Date)
subadult male	11 (−33.7)	YRQ	2010-05-23 (Birth Date)
Total		11	

**Table 2:** Description of the pattern of interference in Tibetan macaques.

Pattern	Definition
Copulation behavior	A male mounting a female with intromission and penis thrusting, with or without ejaculation [31,32].
Ejaculation	Ejaculate visible on the genitalia or perineum of the male or female after the pair dismounted or when the male ceases intravaginal thrusting, shows muscular body spasms and rhythmical pants and has a frowning, round mouth expression [31,32].
Pre-ejaculatory phase	The period in which copulation began when a female was mounted by a male to the time of ejaculation [31,32].
Post-ejaculatory phase	The period following ejaculation, ending when the male’s penis was removed from the female’s vagina [31,32].
Interruption	A individual approaches a copulating pair (a mating male or a mating female) with aggressive contact (e.g., slapping, touching, grabbing), that separates the copulating pair [31,32].
Harassment	An individual approaches a copulating pair (a mating male or a mating female) with vocalizations, facial expressions, or rarely with aggressive contact (e.g., slapping, touching, grabbing) that does not end in separation of the copulating pair prior to ejaculation [31,32].
Approach	Any movement into the 1m radius of a copulating pair regardless of the patterns of interference behavior [31].
Facial expressions	Lip-smacking and bared-teeth display (e.g., closed-mouth and bared-teeth face, and open-mouth and bared-teeth face directed toward the mounter or mountee) [31,32].
Contact	Any physical contact between an outsider and a copulating pair (e.g., clasping, slapping, touching the head of a mating male and a female) [31,32].
Performer	Adult or subadult male who performed interference behaviors within the distance of 1 m of a copulating pair, and the actor has a social interaction (affiliative or agonistic behaviors) directed at the copulating pair [31,32].
Receiver	Mating male or mating female was harassed or interrupted within 1 m of the sexually-interfering individual(s) [31,32].

## Data Availability

The data presented in this study are available on request from the corresponding author.
